# Prevalence and determinants of erectile dysfunction among diabetic patients attending in hospitals of central and northwestern zone of Tigray, northern Ethiopia: a cross-sectional study

**DOI:** 10.1186/s12902-017-0167-5

**Published:** 2017-03-15

**Authors:** Awole Seid, Hadgu Gerensea, Shambel Tarko, Yosef Zenebe, Rahel Mezemir

**Affiliations:** 1 Department of Nursing, Aksum University, Aksum, Ethiopia; 2 Department of Psychiatric Nursing, Aksum University, Aksum, Ethiopia; 3 Department of Surgical Nursing, St. Paul Medical College, Addis Ababa, Ethiopia; 4 Po.box. 1010, Aksum, Ethiopia

**Keywords:** Prevalence, Erectile dysfunction, Determinants, Diabetes Mellitus, Tigray, Ethiopia

## Abstract

**Background:**

The prevalence of erectile dysfunction among diabetic men varies between 35–90%. Although erectile dysfunction is widespread among men with diabetes, the condition often remains undiagnosed and demands appropriate assessment and prompt treatment. Erectile dysfunction can affect all aspects of a patient’s life including physical, emotional, social, sexual, and relationships. The main aim of this study is to determine the prevalence and determinants of erectile dysfunction among diabetic patients attending hospitals in the Central and Northwest zone of Tigray, Ethiopia.

**Methods:**

A hospital based cross-sectional study was conducted on 249 male diabetic patients attending five hospitals in the Central and Northwestern Zone of Tigray, Ethiopia using systematic random sampling. The data was collected from January 1 – February 30, 2016 and was entered and analyzed using SPSS version 20. Correlation and multivariate logistic regression was employed to test associations between independent and outcome variables.

**Results:**

The mean age of study participants was 43.39 years and the mean duration of diabetes diagnosis was 6.22 years. The overall prevalence of erectile dysfunction was 69.9%, with 32.9% suffering from mild, 31.7% moderate, and 5.2% severe erectile dysfunction. Multivariate logistic regression revealed that erective dysfunction was significantly predicted by old age (Adjusted Odds Ratio [AOR] =15.013, CI:3.212–70.166), longer duration of diabetes (AOR = 3.77, CI:1.291–11.051), and lower monthly income (AOR = 0.285, CI:0.132–0.615). No association was found with body mass index, co-morbidity, glycemic control, and alcohol consumption.

**Conclusion:**

The prevalence of erective dysfunction in this study population was very high. Age, income, and duration of diabetes were the independent predictors of erectile dysfunction. Nearly all of the patients in the sample (97%) had not been screened or treated for erectile dysfunction. Assessment and management of erectile dysfunction in the diabetic clinic should be part of routine medical care during follow-up visits with diabetic patients. Healthcare providers should put an emphasis on screening and treating older patients and those who had a diabetes diagnosis for a longer duration.

**Electronic supplementary material:**

The online version of this article (doi:10.1186/s12902-017-0167-5) contains supplementary material, which is available to authorized users.

## Background

Erectile dysfunction (ED), or impotence, can be defined as an inability to initiate and have a persistence erection firm enough to have satisfying sexual intercourse. In relation to the increasing prevalence of chronic conditions, such as Diabetes Mellitus (DM), chronic complications such as ED are also rising [1]. The rate of occurrence of ED in men with DM is two to three times higher than in men without DM. Moreover, men with diabetes may experience ED as much as 10 to 15 years earlier than men without diabetes [2].

Studies conducted on prevalence rates of ED among diabetic men produce various results which generally range from 35–90%. For instance, ED rates among diabetic men were over 50% in the United States, 35–78% in Mexico, 41% in the Netherlands, 80-90% in Saudi Arabia, and 77% in the Isfahan province of Iran [3, 4]. Moreover, studies have demonstrated high rates of ED in Africa. The prevalence of ED is 72.5% in Nigeria and 55.1% in Tanzania, with 12.8% of participants suffering from mild dysfunction, 11.5% from moderate, and 27.9% from severe dysfunction [5, 6].

Sexual function is an important indices of quality of life. The presence of ED has been negatively associated with men’s social interactions, emotional and psychological well-being, and partner relationships’. It is important to note that ED is one of the most treatable complications of diabetes; the literature demonstrated that over 95% of cases can be successfully treated [7].

Further complicating the issue is the sensitivity of the disorder. Erectile dysfunction is an issue that requires privacy; many men feel embarrassed to disclose and discuss the problem with their doctor, or even their life partner. In Ethiopia, open discussions about sexual matters occur infrequently, making the issue of privacy even more important. There is currently a gap in the literature, as no studies of ED have previously been conducted in Ethiopia. Thus, this study will provide evidence regarding the sexual health, specifically ED, of diabetic patients and will contribute to efforts to improve the quality of life of diabetic patients including the prevention of undesirable psychosocial consequences.

## Methods

### Design

A hospital based cross-sectional study was conducted on 249 male diabetic patients attending five hospitals in the Central and Northwestern Zones of Tigray located in northern Ethiopia. The study hospitals are St. Mary’s Hospital (Aksum), Adwa Hospital, Suhul Hospital (Shire), May Ayni Hospital (Shiraro), and Abi Adi Hospital. These hospitals were selected due to their accessibility and affiliation with Aksum University. The data was collected from January 1 – February 30, 2016 for two consecutive months. Ethical clearance was obtained from the Institutional Review Board (IRB) of Aksum University College of Health Sciences. A letter of support was also issued from the Tigray Regional Health Office to each respective health institution. In addition, information about the study was provided and informed consent was obtained from study participants to confirm their willingness for participation. To enable participants to disclose and respond openly, five data collectors who are male clinical nurses working at each respective study hospital were recruited. Moreover, to maintain privacy, anonymous questionnaires were used and patients were interviewed alone and by the male clinical nurses.

### Recruitment and Sample

A systematic random sampling technique was used to identify participants. Adult male patients age ≥ 18 years with a diagnosis of DM were included in the study. The diagnosis of DM was made by physicians (internists) working at each study hospital. We utilized the World Health Organization criteria of a Fasting Blood Sugar (FBS) ≥126 mg/dl to diagnose a patient with DM. Fasting blood sugar was measured using a glucometer immediately before breakfast when the patient arrived at the diabetic/chronic disease follow-up clinic of each respective study hospital. Study participants with known secondary ED from genetic, endocrine, neurological, or surgical causes were excluded from the study.

### Measures

To assess for erectile function a pre-test and structured interviewer administered questionnaire adopted from the abridged 5-item version of the International Index of Erectile Function (IIEF-5) was used [8]. Data were gathered from both the chart and self-report of patients. The tool also included socio-demographic variables, clinical characteristics, and other variables. It was translated to the local language (Tigregna) and back translated to English by professional translating organizations (Additional file 1). Clinical characteristics of the patients were also measured including Body Mass Index (BMI) and blood pressure. A manual sphygmomanometer (blood pressure cuff) was used to measure blood pressure and the World Health Organization criteria were used to classify BMI.

### Operational definitions

The following operational definitions were used for the outcome variable of ED based on the scores using the International Index of Erectile Function (IIEF-5).

- **Severe ED**: Study participants who scored 1–7 out of 25 points.
- **Moderate ED**: Study participants who scored 8–11 out of 25 points.
- **Mild to Moderate ED**: study participants who scored 12–16 out of 25 points.
- **Mild ED**: study participants who scored 17–21 out of 25 points [8].

### Data analysis

The sample size was calculated using a single proportion formula considering 95% confidence interval and 5% margin of error. Since there were no previous studies conducted in Ethiopia, we utilized the prevalence studied in Nigeria as *p* = 41.5% [9]. The sample size was reduced by using the population correction formula and by adding 10% for non-response rate to result in the final sample size of 249. Finally, proportional sample size was taken from each five hospitals as per the total population. The study subjects were selected using systematic random sampling (*k* = 2) at diabetic and chronic disease follow-up clinics. The collected data were entered and analyzed using SPSS version 20; *p* values were set at *p* < .05. Texts, charts, and tables were used to describe the result. Pearson correlation and multivariate logistic regression were used to analyze the association of independent and outcome variables.

## Results

### Socio-demographic characteristics of participants

Two hundred forty nine (249) patients were interviewed to achieve the study aim with a 100% response rate. The mean age of study participants was 43.39 ± 14.7 years (range: 19–83 years). The mean duration of a DM diagnosis was 6.22 years (range: 1 month – 30 years). Almost all (98.4%) of the participants were of Tigre ethnicity and the majority (86.7%) were also married. Participants were also predominantly of the Orthodox religion (88%) and had low educational status. Most participants were farmers (49.8%), followed by government employees (19.3%). As depicted in Table 1, a substantial proportion (43.4%) of respondents had a monthly income of lower than $25.

**Table 1:** Socio-demographic characteristic of participants in Tigray, Ethiopia, 2016

Variable	Frequency (Percent)
Age	
<30	63 (25.3)
30–44	75 (30.1)
45–59	65 (26.1)
>60	46 (18.5)
Ethnicity	
Tigre	245 (98.4)
Others	4 (1.6)
Marital status	
Single	24 (9.6)
Married	216 (86.8)
Separated	1 (0.4)
Divorced	6 (2.4)
Widowed	2 (0.8)
Religion	
Orthodox	219 (88.0)
Muslim	27 (10.8)
Others	3 (1.2)
Educational status	
Illiterate	59 (23.7)
Grade 1–8	117 (46.9)
Grade 9–12	32 (12.9)
College/university	41 (16.5)
Occupational status	
Farmer	124 (49.8)
Government employee	48 (19.3)
NGO employee	24 (9.7)
Merchant	22 (8.8)
Daily labor	16 (6.4)
Others	15 (6.0)
Monthly income ($)	
<25	108 (43.4)
25–50	67 (26.9)
>50	74 (29.7)

### Clinical and life style characteristics of participants

The participant’s life style factors demonstrated that 40.6% drink alcohol, 2.8% smoke cigarettes, and 81.9% did not engage in regular exercise. Clinical characteristics of the participants indicated that the mean FBS level of study participants was 187.5 mg/dl, with 35.3% of participants in the normal range (<126 mg/dl) and 64.7% of participants above the normal range (≥126 mg/dl) implying that the majority of participant’s had poor control of their glycemic status at the time the study was conducted. Additionally, the mean BMI of respondents was 20.77 kg/m^2^ (range: 13.8-14.9 kg/m^2^). Results indicated that 71.5% of study participants were in normal range for BMI (BMI = 18.5 – 24.9 kg/m^2^), 19.3% were underweight (BMI < 18.4Kg/m^2^), and 9.2% were overweight (BMI = 25-29.9Kg/m^2^). No obese patients (BMI > 30 kg/m^2^) were found in this study. The majority of participants (90.4%) were within the normal range for blood pressure (BP < 120/.90) while the rest of participants had either Stage 1 (8.4%) or Stage 2 (1.2%) hypertension. Forty participants (16.7%) had additional chronic disease beside DM. The most common chronic disease co-existing with DM was hypertension which accounted for 9.2% of cases. The breakdown for type of DM was 63.1% were Type 1 and the remaining 36.9% were Type 2 DM (Table 2).

**Table 2:** Cross tabulation clinical variables with presence of erectile dysfunction in diabetic patients attending hospitals in the central and northwestern zones of Tigray, Ethiopia, 2016

Clinical variables	Erectile dysfunction	No erectile dysfunction	Row total
Type of DM			
Type 1	86 (67.2-)	42 (32.8-)	128
Type 2	64 (76.2-)	20 (23.8-)	84
BMI			
Under weight	27 (65.9)	14 (34.1)	41
Normal	107 (72.3)	41 (27.7)	148
Over weight	16 (69.5)	7 (30.4)	23
Obesity	0 (0.0)	0 (0.0)	0
Blood pressure			
Normal range	131 (69.3)	58 (30.7)	189
Stage 1	17 (85.0)	3 (15.0)	20
Stage 2	2 (66.6)	1 (33.4)	3
Duration of diabetes (years)			
<5	80 (66.1)	41 (33.9)	121
5–10	38 (70.3)	16 (29.7)	54
>10	32 (86.5)	5 (13.5)	37
Smoker			
Yes	5 (71.4)	2 (28.6)	7
No	145 (70.3)	60 (29.7)	205
Physical exercise			
Yes	24 (60.0)	16 (40.0)	40
No	126 (73.3%)	46 (26.7)	172
Drink alcohol			
Yes	66 (70.9)	27 (29.1)	93
No	84 (70.6)	35 (29.4)	119
FBG (mg/dl)			
<126	56 (74.6)	19 (25.4)	75
≥126	94 (68.6)	43 (31.4)	137

### Prevalence and determinants of erectile dysfunction (ED)

**Fig. 1:**
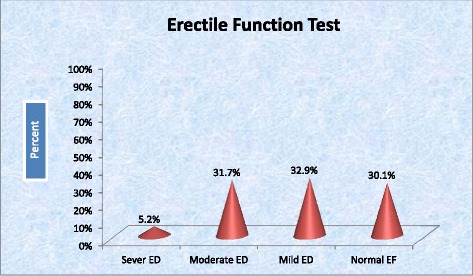
Distribution of severity of erectile dysfunction among diabetics attending at central and northwest Tigray, 2016

The mean score on the International Erectile Function Test (ILEF-5) was 13.86 ± 4.375 (range: 5–25). The prevalence of ED in this study was found to be 69.9% of which 5.2% had severe ED, 31.7% had moderate ED, and 32.9% had mild ED. Only 30.1% of patients had normal erectile function (Fig. 1). More specifically, the prevalence of ED in Type 1 and Type 2 DM patients was 60.2 and 76.2% respectively.

When evaluating treatment seeking behavior, only 8 (3.2%) of patients sought medical care because of ED. Relatedly, among those patients who had been treated for ED, only one respondent was treated by both pharmacotherapy and psychotherapy.

Multivariate logistic regression analyses show that there was an association between ED and age, income, and duration of diabetes. Respondents whose age was above 60 years old were 15 times more likely to develop ED as compared to those less than 30 years of age (AOR = 15.013, CI:3.212 – 70.166, *p* = .001). Moreover, study participants who had a diagnosis of DM for more than 10 years were 3.77 times more likely to develop ED as compared to those who had a diagnosis of diabetes for less than 10 years (AOR = 3.77, CI:1.291-11.051, *p* = 0.015). On the other hand, study participants whose monthly income was above $50 were 0.249 less likely to have ED as compared to those with an income below $25 (AOR = 0.285, CI: 0.132-0.615 *p* = .001). Otherwise, no associations were found between ED and BMI, co-morbidity, glycemic control, and alcohol consumption (Table 3).

**Table 3:** Multivariate logistic regression of factors affecting erectile function of diabetic patients in the central and northwestern zones of Tigray, Ethiopia 2016

Factors	AOR	95% CI	*P* value
Age			
<30 year	1.0	-	-
30–48 year	1.028	0.499 – 2.120	.940
49–60 year	2.09	0.903 – 4.846	.085
>60	15.01	3.212 – 70.166	.001*
Income ($)			
<25	1.0	-	-
25–50	0.486	0.233–1.012	.054
>50	0.285	0.132–0.615	.001*
Duration of diabetes			
<5 year	1.0	-	-
5–10 years	1.058	0.538–2.080	.871
>10 years	3.778	1.291–11.051	.015*
Co-morbidity			
No	1.0	-	-
Yes	1.534	0.649–3.628	.329

*Significant association at *p* < 0.05

## Discussion

In this study, the majority (74.7%) of Type 1 DM patients were above the age of 30 years. On the other hand, Type 1 DM is more common than Type 2 DM in the Central and Northern Zones of Ethiopia. This might be attributed to the fact that the greatest risk factor for Type 2 DM is obesity, which is largely absent in the Central and Northern Zones of Ethiopia. The study region is similar to other regions of the country and thus results should be considered as they may pertain to the country as a whole.

The study confirms that the prevalence of ED among DM patients is high (69.9%) of which 5.2% had severe ED that required immediate intervention. The high prevalence of ED among DM patients is in line with several studies. The prevalence of erectile dysfunction among diabetic men which has been reported across the world varies between 35–90%. The result of this study are within this range. Moreover, the study finding coincide with the results from Nigeria in which the prevalence of ED is higher in diabetics than in other chronic diseases which reaches up to 72.7%. In another study in Iran, sexual dysfunction was detected in 77% of diabetic men, and in Saudi Arabia the prevalence ranges from 80–90%. However, there is some discrepancy with the study conducted at Tanzania, and Iran which is 55 and 59.5% respectively. The reason may be attributed to differences in the socio-cultural context of study participants and the methodology. In this study, the chart review and in-person interviews may have uncovered additional cases of ED that would not have been found with only one data collection method. Beside this, in the United States the prevalence of ED among the general population is 18.4% versus 51.3% in diabetic population. But the data collection tool used in this study was a one item question which is very different from this study [10].

A review of current perspectives on diabetes and sexual dysfunction show the prevalence of ED is reported ≥ 50% in male with diabetes worldwide which is congruent with the results of this study. Past literature has also demonstrated that advanced age, longer duration of diabetes, co-morbidity with hypertension, hyperlipidemia, glycemic control, smoking, sedentary lifestyles, being overweight, and obesity are the common independent predictors for ED. However, this study demonstrates that only advanced age, longer duration of diabetes, and lower income were significantly associated with ED in Ethiopia. This might be due to discrepancy of methodology, data collection tools, the sample size, and socio-economic differences of study participants [11]. On the other hand, a study conducted in Italy and Israeli men on erectile dysfunction in Type 1 and Type 2 DM demonstrates similar finding on the independent predictor variables [12, 13].

The study further confirms that duration of diabetes, age, and monthly income are the independent predictors for ED and its severity. In contrast, this study reveals BMI, glycemic control, co-morbidity, taking concomitant drug are not associated with ED. Most studies have similar results demonstrating age and duration of diabetes are significantly associated with ED. Older age groups, lower income, and patients with long duration with DM are more likely to develop ED. Even the AOR (AOR = 15) result among age groups above 60 years of age is similar with Tanzania. Several reasons including age related physiological changes in the testicles and decline in male sex hormones have been attributed to the increasing incidence of ED in older men [5]. On the other hand, obesity, glycemic control, co-morbidity, and taking additional drugs and drinking alcohol were not associated with ED. But in some studies these variables have been associated [4, 14].

Among the 166 patients in this study who had ED only eight sought medical help in order to be treated for the disease. Almost all patients (97%) had not been screened or treated for ED. This disparity might be attributable to patients’ feeling embarrassed, not considering ED a treatable disease, and fear of side effects.

## Conclusion

It is important to consider DM as a threat for rural communities. The prevalence of ED in the study population was very high. The prevalence of ED among Type 2 DM patients was relatively higher than Type 1 DM. Almost all patients (97%) had not been screened or treated for ED. Increasing age, relatively lower income, and longer duration of DM were significant predictors of ED.

Assessment and management of ED should be part of routine medical care in diabetic follow-up clinics. Healthcare providers should openly ask men with chronic disease, particularly those with diabetes, about symptoms of ED. The medications used for treatment should be readily available in the hospital settings in Ethiopia. Mental health professions should also be involved in providing psychotherapy (sexual counseling) for patients with ED. Patients who are of older age and who have a longer duration of DM require special attention in screening for ED.

## Additional file

Additional file 1: Questionnaire used to collect erectile dysfunction of diabetic patients. (PDF 154 kb)

## Abbreviations

BMI: Body mass index
DM: Diabetes mellitus
ED: Erectile dysfunction
FBG: Fasting blood glucose
IIEF: International index of erectile function
