# Supplementary material for: Prevalence and determinants of erectile dysfunction among diabetic patients attending in hospitals of central and northwestern zone of Tigray, northern Ethiopia: a cross-sectional study
Source: BMC Endocr Disord. 2017 Mar 15;17:16. doi: 10.1186/s12902-017-0167-5 (PMC5353861; doi:10.1186/s12902-017-0167-5)
Supplement: Additional file 1: — Questionnaire used to collect erectile dysfunction of diabetic patients. (PDF 154 kb) [file 12902_2017_167_MOESM1_ESM.pdf]

Annex: I Questionnaire (English Version) No_________

Aksum University

College of Health Sciences

This questionnaire is designed to determine Prevalence and Determinants of Erectile Dysfunction among Diabetic Patients attending in Hospitals of Central and Northwestern Zone of Tigray, Northern Ethiopia.

Direction for Data Collectors: please circle the patients' response from the given options.

1. **Socio-demographic characteristics**

1.1. **Age** (year) ________

1.2. **Ethnicity** 1. Tigray 2. Other(s) _______________

1.3. **Marital Status**

1. Single 2. Married 3. Separated
4. Divorced 5. Widowed

1.4. **Religion**

1. Orthodox
2. Muslim 3. Protestant 4. Other (s) _____________

1.5. **Educational Status**

1. Illiterate
2. Primary (Grade 1-8)
3. Secondary (Grade 9-12)
4. College/university

1.6. **Occupational Status**

1. Farmer 4. Merchant
2. Gov't employee 5. Daily Laborer
3. NGO employee 6. House wife 7. Other(s) _________

1.7. **Monthly Income (ETB)**

1. ≤ 500 2. 501-1000 3. ≥1001

II. Life Style Habits

1. Do you drink alcohol? 1.1. No 1.2. Yes

   If yes Type_______________

   Amount ____________

2. Do you smoke cigarettes? 1.1. No 1.2. Yes

   If yes! Number of packets per day____________

3. Do you exercise regularly? 1.1. No 1.2. Yes
4. BMI of the patient

   Height (meter) _____________

   Weight (K.g) _______________ BMI ____________ K.g/m^2

5. Blood Pressure of the patient__________mmHg.
6. Current Fasting Blood Sugar (FBS) of the patient____________ mg/dl

III. Medical Characteristics

1. Do you have chronic diseases other than DM? 1.1. No 1. 2. Yes
2. If yes to Q.1 what is (are)

   2.1. Hypertension

   2.2. Congestive Heart Failure

   2.3. Chronic Kidney Disease

   2.4. Chronic Liver Disease

   2.5. Rheumatoid Arthritis

   2.6. Tuberculosis

   2.7. Other (specify)____________________

3. How long you have been diagnosed with DM? _______________Months/years.
4. Which type DM you have? 4.1. Type 1 4. 2. Type 2
5. Do you take any drugs other than for DM? 1.1. No 1.2. Yes (specify)___________

IV. The IIEF-5 Questionnaire

S. No	Over The Past Six Months:					
1	How do you rate your Confidence that you could get and keep an erection?	Very low 1	Low 2	Moderate 3	High Very 4	High 5
2	When you had erections with Sexual stimulation, how often were your erections hard enough for penetration?	Almost never/never 1	A few times (much less than half the time) 2	Sometimes (about half the time) 3	Most times (much more than half the time) 4	Almost always/always 5
3	During sexual intercourse, how often were you able to maintain your erection after you had penetrated (entered) your partner?	Almost never/never 1	A few times (much less than half the time 2	Sometimes (about half the time) 3	Most time (much more than half the time) 4	Almost always/ always 5
4	During sexual intercourse, how difficult was it to maintain your erection to completion of intercourse?	Extremely difficult 1	Very difficult 2	Difficult 3	Slightly difficult 4	Not difficult 5
5	When you attempted sexual intercourse, how often was it satisfactory for you?	Almost never/never 1	A few times (much less than half the time) 2	Sometimes (about half the time) 3	Most times (much more than half the time) 4	Almost always/always 5

V. Acceptance and Treatment Seeking rate for ED

1. Have you ever seek any medical help for sexual related problems before?
    1. No 2. Yes
2. If 'yes' did you treated for the sexual problem?
    1. No 2. Yes (drug/psychotherapy)
3. Do you think that drugs are effective in resolving sexual problems in diabetics?
    1. No 2. Yes
4. Do you think sexual counseling is effective in resolving sexual problems in diabetics?
    1. No 2. Yes

Thank you for your cooperation!!!

Data Collector Name ____________________________ ***Name of the Hospital*** _______________

Signature ____________________ ***Date*** ______________________
